# Centennial—American Medical Association and Convention of Medical Colleges

**Published:** 1876-07

**Authors:** 


*Centennial—American Medical Association and Convention of Medical Colleges—Extract from the Letter of a Medical Friend.*— By the way, in your visit to the Centennial, did you see and note anything of special interest? If so, will you not give us some account of the same in the *Record*? Did you attend the *American Medical Association* at its late meeting? In all deference to you and to other good men who are connected with that body, permit me to say that in my humble judgment it has failed to meet the expectation of the profession as to the accomplishment of anything good. It seems to be little else than a medium by which a few men obtain notoriety for themselves. It is stated in the public prints that Dr. Sims, the President, denounced the ethics in his address before the Association. Can this be so? What did the Convention of Medical Colleges do?

OUR VISIT TO PHILADELPHIA.

It was our intention to publish some account of our late visit to the Centennial, even though the above letter had not been received. But we are glad that our friend has made the inquiries and are gratified for the opportunity of answering his letter in our leader upon this subject.

To describe all that a visitor sees at the Centennial would be impossible. We will attempt only an outline of what we saw at that world's wonder. It is the aggregation of the results of progressive art, genius and intellect of the last century, and we may safely say eclipses all displays of the kind ever before made in any portion of the world.

Here may be seen the product of nearly every country upon the globe, presided over by its own citizens, in their national costume and speaking their mother tongue. Until they see them here grouped in one grand and beautiful whole, even men of the most extensive information cannot realize the vast amount of industry that produces so great a variety of articles from the busy marts of the civilized world. It has been truly said, that a visit to this wonderful exhibition would teach a youth, and even a man, more of natural history, the triumphs of agriculture, and mechanical genius, and the marvels of beauty and art, than he would learn from schools or books in a lifetime.

THE EXHIBITION GROUNDS

Are of vast extent, within an enclosure of 256 acres, and are fitted with all appliances for pleasure, comfort and convenience of the visitor. There is a telegraph office and a national bank in the enclosure, and the grounds are ornamented with lakes and fountains, rare trees and shades, vases and statuary.

MAIN EXHIBITION BUILDING.

This is 1880 feet by 664 feet. Upon the corners of the buildings there are four towers, 75 feet in hight, from which a fine view can be obtained of the grounds and the city. In this building are the departments of mining and metallurgy, manufactures, education and science. This edifice cost $1,420,000, exclusive of drainage, water-pipe, fountain, etc.

THE ART GALLERY,

Also called Memorial Hall, and intended to be a permanent structure, is a thoroughly fire-proof building of granite, glass and iron, and was erected at the cost of $1,500,000. It is 365 feet long and 210 feet wide, surmounted by a beautiful central dome. Here the wonderful and beautiful conceptions of Raphael, Rubens, Correggio and a noble array of others, equally gifted, look down upon you from the lofty walls. The immortal *genius* of our own artists, displayed in their productions on the opposite side, seem to challenge the other for the prize of excellence upon the seemingly living canvas, and in the almost breathing marble.

MACHINERY HALL.

From art and beauty to the eminently useful and practical, we are now within this building, with its immense floor capacity, its eight lines of shafting, and great Corliss engine of 1,400 horse power, capable of putting in motion the fifteen acres of machinery of all motions. Here the visitor can see manufactured upon the spot foreign sample productions, among them Persian and Turkish rugs, carpets, pins, needles, confectioneries, etc. Fifteen nations, besides the United States, are represented in this building.

### HORTICULTURAL HALL.

From the deafening clatter of this wilderness of machinery, we are back to the beautiful again in Horticultural Hall. The city of Philadelphia contributes liberally to the expense of this building, and it is intended as a permanent ornament to the park. It is built in the Moorish style of architecture of the twelfth century, the external materials being principally iron and glass, and is 333 feet long and 193 feet wide. The central conservatory has a height of 55 feet, surmounted by a lantern 170 feet long, 20 feet wide and 14 feet high. This is a favorite building for the ladies, and all lovers of the beautiful. To enter it by its flights of blue marble steps, is like treading upon enchanted grounds. One is unconsciously borne back to gorgeous scenes in the Arabian Nights, but feels that the genii of Aladdin's Lamp could never have summoned the magical and bewildering beauty of this hall, when lighted with its 3,500 gas burners. Nature and art, with harmonious action, seem to have exerted almost miraculous power in attaining such perfection and marvelous beuuty as is displayed in this magnificent bower of bloom, and the sweet incense of the floral world.

### AGRICULTURAL HALL.

A visit to this building is interesting to all persons, but especially to the farmer and scientific agriculturalist. Here is a display of all the products of the forest, both in a primary and secondary form, and the fruits of our varied climates, with those of more Northern and tropical regions. The exhibits though in pomology will necessarily be larger towards the latter part of August or September.

The Chemical and Pharmaceutical Department is exceedingly interesting and instructive. "Nearly all nations are represented." We were proud to see that our chemists are equal, if not superior, to those of any country on the globe. In purity and neatness our manufacturers are far superior to all others. We are almost independent in this department. The most prominent exhibitors are Messrs. Powers & Weightman, of Philadelphia; Messrs. Chas. T. White & Co., of New York; Harrison & Brothers & Co., of Philadelphia, and Charles P. Pfiser & Co., of New York. Messrs. John Wyeth & Brother, of Philadelphia; Messrs. Rosengarten & Son, of Philadelphia, and Messrs. Billings, Clapp & Co., of Boston—it is said they present the largest number of specimens.

There is also a fishery with a hatching apparatus, showing the manner of fish culture. The display of different woods is very fine: the rose, ebony and mahogany, of Brazil, and the products in this line from Spain and Portugal are handsomely displayed, while the Japanese exhibits are curious and instructive. Of course there are cereals of all kinds, in fact every farm product, implement and machinery for the farm. Some of these implements are finished very highly. One set of plows from Indiana appear in gold plating, at a cost of $1,000 each. Though there are exhibited every kind of cotton grown in the world, the display is small. Only Egypt and Brazil exhibit samples of foreign cotton.

RESTAURANTS.

Are handsome and commodious; but we have space to mention only one or two. One of the most unique upon the grounds is that put up by Mr. Edward Mercer, of Atlanta. The structure is ornate and capacious—the dining room seating 500 guests at once, and its sensation novelty, especially to the foreign nations, is a band of old-time plantation negroes with their unique songs, the strumming of the banjo and "rattling of the bones." Mr. Mercer's establishment is called "The South.' 'The principal French restaurant is in charge of Leon Goyard, Paris, and has a beautiful outlook upon the lake. Leon Goyard personally superintended the official dinners given by the emperor of Austria, and had charge of all the dinners given to the sovereigns who visited the Vienna Exhibition of 1875.

But our space forbids us to give anything like an elaborate description of this exhibition. The foregoing brief sketch may be of some interest to our readers who cannot attend the Centennial, or something of a foretaste of the feast that those may expect, who are so fortunate as to look upon this grand assembly of representative products, and citizens of the civilized world.

